# Provider-Initiated HIV Testing in Puerto Rico from Data of the National HIV Behavioral Surveillance–Heterosexual Cycle (NHBS-HET) 2016: National Cross-sectional Survey

**DOI:** 10.2196/29890

**Published:** 2022-10-26

**Authors:** Vivian Colón-López, Derick Pérez-Guzmán, Maureen M Canario De La Torre, Nadia Centeno-Alvarado, Ivony Y Agudelo-Salas, Yadira Rolón, Sandra Miranda, Maria Pabón, Jorge Rodríguez-Lebrón, Gladys Girona Lozada

**Affiliations:** 1 Cancer Control and Population Sciences Program Comprehensive Cancer Center University of Puerto Rico San Juan Puerto Rico; 2 Evaluation Program, Health Services Administration Graduate School of Public Health Medical Sciences Campus, University of Puerto Rico San Juan Puerto Rico; 3 Data Analysis and Publication Core Comprehensive Cancer Center University of Puerto Rico San Juan Puerto Rico; 4 HIV/AIDS Surveillance Program Puerto Rico Health Department San Juan Puerto Rico

**Keywords:** HIV, HIV testing, heterosexual, health care provider, Puerto Rico, National HIV Behavioral Surveillance, NHBS, behavioral surveillance system, HIV infection, Centers for Disease Control and Prevention, CDC, respondent-driven sampling

## Abstract

**Background:**

According to the Centers for Disease Control and Prevention and World Health Organization guidelines, all individuals aged 13-64 years should get screened for HIV infection as part of their routine medical examinations. Individuals at high risk should get tested annually.

**Objective:**

This study aimed to identify the sociodemographic, health care, and sexual behavioral characteristics of provider-initiated HIV testing using data from the Puerto Rico National HIV Behavioral Surveillance 2016 cycle, directed toward heterosexual individuals at increased risk of HIV infection.

**Methods:**

A sample of 358 eligible participants were recruited through respondent-driven sampling, where sociodemographic characteristics, health care use, and HIV test referral were used to assess a description of the study sample. Pearson chi-square and Fisher tests were used to evaluate proportional differences. Multivariate logistic regression models were performed to determine the association between independent variables and HIV test referral. Adjusted prevalence ratios by sex and age with their 95% CIs were determined using a statistical significance level of .05.

**Results:**

Despite 67.9% (243/358) of participants showing high-risk sexual behavioral practices and 67.4% (236/350) reporting a low perceived risk of HIV infection among those who visited a health care provider within the last 12 months, 80.7% (289/358) of the study sample did not receive an HIV test referral at a recent medical visit. Multivariate analysis showed that the estimated prevalence of the participants who received an HIV test referral among those who reported being engaged in high-risk sexual behaviors was 41% (adjusted prevalence ratio .59, 95% CI .39-.91; *P*=.02) lower than the estimated prevalence among those who did not engage in high-risk sexual behavior.

**Conclusions:**

This sample of Puerto Rican adults reported a significantly lower prevalence of receiving an HIV test referral among heterosexual individuals at increased risk of HIV infection who engaged in high-risk behaviors. This study further emphasizes the need for health care providers to follow recommended guidelines for HIV test referrals in health care settings. Promotion practices in the future should include enhancing referral and access to HIV tests and implementing preventive measures to counteract the HIV epidemic in Puerto Rico.

## Introduction

Heterosexual contact is the second most common route of HIV transmission in the United States [1]. Data from the Puerto Rico HIV Surveillance System reported that heterosexual contact was the most prevalent mode of HIV transmission, accounting for 36% of the total cases in 2016. The vast majority (70%) of HIV-diagnosed cases were men. However, among heterosexual individuals, women were the most affected by this transmission route, accounting for approximately 62% of those cases [2]. In Puerto Rico, studies have shown a high prevalence of high-risk sexual behaviors among men and women [3], where more than half (60%) of heterosexual men and women have ever been tested for HIV in their lifetime (unpublished Puerto Rico National HIV Behavioral Surveillance [NHBS] data, 2010 cycle).

According to the 2006 guidelines from the Centers for Disease Control and Prevention (CDC) on HIV testing, all individuals aged 13-64 years should be screened for HIV infection as part of their routine medical examinations. Individuals at high risk should get tested annually [4]. The World Health Organization and the Joint United Nations Program of HIV/AIDS recommend that health care providers offer patients opt-out HIV testing and counseling. Specifically, it is suggested that HIV testing be offered to patients regardless of the presence or absence of clinical symptoms of HIV infection and the patient’s motive(s) for seeking health advice [5]. This effort, known as provider-initiated HIV testing (PIHT), has resulted in an increased number of patients being tested [6,7].

Despite the previous recommendations, the rigorous application of this practice is not seen in most health care scenarios [8]. Previous studies have identified barriers that might account for the lax implementation of PIHT [9,10]. Some barriers include the lack of time during routine visits, the health care provider’s beliefs that their patients would feel uncomfortable discussing HIV infection, a patient’s idea that their previous results (before the last 12 months) remain valid, and the provider’s (or patient’s) sense that such a conversation would be inappropriate because of a poor doctor-patient relationship [7-11]. However, further studies have reported that such barriers could be overcome through provider initiatives such as adopting a sensitive attitude when engaging patients in discussing HIV testing and making educational reinforcement on HIV [12,13].

The number of individuals tested for HIV in 2010 still falls short of the expected number [14]. The burden of HIV infection in heterosexual individuals at high risk in Puerto Rico and the current low–HIV testing uptake highlights the need for understanding the correlates and characteristics of PIHT. This study aimed to identify the sociodemographic, health care, and sexual behavioral characteristics of PIHT using data from the Puerto Rico–NHBS. Insights into PIHT profile may help develop strategies for the early identification of new HIV cases, contributing to better patient care and outcomes.

## Methods

### Data Source

The NHBS is a cross-sectional survey performed in the United States and its jurisdictions that collects data related to high-risk behavioral practices for HIV infection in 3 different populations: men who have sex with men, persons who inject drugs, and heterosexual individuals at increased risk of HIV infection [15]. NHBS procedures and methods can be found elsewhere [15]. The NHBS performs consecutive surveys in each cycle, in which the abovementioned populations are studied separately in rounds composed of independent cycles.

Participation in this project was completely anonymous and voluntary, which involved completing a face-to-face interview using a standardized questionnaire in Spanish that gathered information about HIV-associated risk behaviors. Additionally, the participants were offered a voluntary HIV test through the interview process, which they could opt out of while still completing the survey. The interviewers described these activities through informed consent to the participants before they began the questionnaire. Participants who completed the NHBS survey and those who agreed to the HIV test received a stipend for their time (US $20 for the survey and US $20 for the HIV test). NHBS staff did not require any personal information from the participants to protect their privacy and confidentiality. Blood specimens and questionnaires were linked by Survey ID numbers only.

### Ethics Approval

The Institutional Review Board of the University of Puerto Rico, Medical Sciences Campus reviewed and approved this study (A0910115).

### Recruitment

Data used for this analysis correspond to the Puerto Rico NHBS–Heterosexual Cycle’s 4th round (PR-NHBS-HET4) conducted in 2016. Recruitment for PR-NHBS-HET4 was performed through respondent-driven sampling (RDS). The implementation of RDS began with a limited number of initial recruits known as “seeds,” which are people who work with the target population and can adequately identify other participants with the ideal characteristics of the NHBS project.

The participating areas were included within the San Juan metropolitan statistical area. The estimated sample size for PR-NHBS-HET4 was 500 participants. Inclusion criteria for seeds and participants were the following: (1) be aged 18-60 years, (2) have had vaginal or anal sex with a person of the opposite sex in the 12 months before the interview date, (3) have not previously participated in any other PR-NHBS-HET cycle, (4) live in 1 of the participating San Juan metropolitan statistical area municipalities, (5) identify as male or female, (6) be able to complete the interview in Spanish or English, (7) have not injected drugs in the 12 months before the interview date, and (8) have a low income as established by the Department of Health and Human Services poverty guidelines or have low educational attainment—no greater than high school education [16,17]. During the PR-NHBS-HET4 cycle, a total of 609 interviews were conducted. Of these participants, 114 were excluded from the study because they did not meet the inclusion criteria, leaving 495 participants—9 of whom were seeds. For this analysis, we restricted the sample to those who visited a health care provider within the last 12 months, leaving 358 participants as the final sample that had complete data on the main outcome of this study.

### Measurements

Variables for sociodemographic characteristics, health care use, HIV testing, and PIHT were assessed per the NHBS core questionnaire [18]. To collect the main outcome data, the participants responded to the question, “At any of those times you were seen [by a doctor, nurse, or other health care provider], were you offered an HIV test?” The participant’s response was dichotomized into 2 categories, “Yes” or “No.” Variables such as educational level, annual household income, employment, and marital status were dichotomized for data analysis. Risk perception was assessed using dichotomized categories (low–risk perception of getting infected with HIV and high–risk perception of getting infected with HIV). A high-risk sexual behavior scale was performed if the study participant reported engaging in at least one of the following in the past 12 months: (1) any exchange of sex for drugs or money; (2) having sex with more than one sexual partner; (3) having sex with a partner who “probably” or “definitely” had other sex partners, concurrently; (4) having sex with a partner who had “probably” or “definitely” injected drugs; (5) having sex with a partner who “probably” or “definitely” had male-to-male sexual contact (only female respondents); and (6) having sex with a partner whose HIV status was “positive” or “indeterminate.”

### Data Analysis

The univariate analysis assessed descriptive measurements for sociodemographic characteristics, high-risk sexual behaviors, and the use of health care services. The bivariate analysis consisted of Pearson chi-square and Fisher exact tests to describe the proportional differences of the variables related to sociodemographic characteristics, HIV testing, high-risk behaviors, and PIHT. To assess the association between selected independent variables and PIHT, a logistic regression analysis was conducted with those variables that showed statistical significance in association with the bivariate analysis. Logistic regression models were performed for each independent variable separately. Crude prevalence ratios and adjusted prevalence ratios (PR_a_) by sex and age are presented with 95% CIs and a statistical significance level established at .05. To estimate PR_a_, we assessed the interaction terms in the logistic model using the likelihood ratio test. All statistical analysis was performed with Stata statistical software (version 17; StataCorp LLC).

## Results

### Sociodemographic Characteristics

Most (250/358, 69.8%) of the sample were women. The respondents’ median age was 39 (SD 12.01) years. The majority (307/358, 85.8%) of the respondents reported having medical insurance at the time of the interview (Table 1).

**Table 1:** Provider-initiated HIV testing in heterosexual individuals at increased risk of HIV infection, Puerto Rico National HIV Behavioral Surveillance Cycle 2016 (N=358).

Characteristic			Total sample, N		Received HIV test referral, n (%)		Did not receive HIV test referral, n (%)		*P* value^a^
All participants			358		69 (19.3)		289 (80.7)		
**Sex (N=358)^b^**									
	Male	108		19 (17.6)		89 (82.4)		.60	
	Female	250		50 (20)		200 (80)			
**Age (years; N=358)**									
	18-29	102		26 (25.5)		76 (74.5)		.06	
	30-60	256		43 (16.8)		213 (83.2)			
**Education (N=358)**									
	High school or less	246		50 (20.3)		196 (79.7)		.46	
	Some college or more	112		19 (17)		93 (83)			
**Employed (N=358)**									
	No	226		38 (16.8)		188 (83.2)		.12	
	Full time or part time	132		31 (23.5)		101 (76.5)			
**Marital status (N=358)**									
	Married or partnered	151		33 (21.8)		118 (78.2)		.29	
	Separated, divorced, widowed, or never married	207		36 (17.4)		171 (82.6)			
**Currently insured (N=358)**									
	No	51		9 (17.7)		42 (82.3)		.75	
	Yes	307		60 (19.5)		247 (80.5)			
**Lacked health care due to cost (N=358)**									
	No	304		63 (20.7)		241 (79.3)		.10	
	Yes	54		6 (11.1)		48 (88.9)			
**Had a usual source of care (N=358)**									
	No	23		3 (13)		20 (87)		.59	
	Yes	335		66 (19.7)		269 (80.3)			
**Ever tested for HIV (N=358)**									
	No	81		4 (4.9)		77 (95.1)		<.001	
	Yes	277		65 (23.5)		212 (76.5)			
**Low–risk perception of getting infected with HIV in the next 12 months (n=350)**									
	No	114		18 (15.8)		96 (84.2)		.40	
	Yes	236		46 (19.5)		190 (80.5)			
**Engaged in high-risk sexual behavior^c^ (N=358)**									
	No	115		29 (25.2)		86 (74.8)		.050	
	Yes	243		40 (16.5)		203 (83.5)			
**Men who engaged in high-risk sexual behavior^c^ (n=108)**									
	No	30		7 (23.3)		23 (76.7)		.33	
	Yes	78		12 (15.4)		66 (84.6)			
**Women who engaged in high-risk sexual behavior^c^ (n=250)**									
	No	85		22 (25.9)		63 (74.1)		.095	
	Yes	165		28 (17)		137 (83)			

^a^Chi-squared test–reported *P* value.

^b^Denominators vary because of nonresponse in the form of missing values.

^c^Defined as engaging in at least one of the following: (1) any exchange of sex for drugs or money; (2) having sex with more than one sexual partner; (3) having sex with a partner who “probably” or “definitely” had other sex partners, concurrently; (4) having sex with a partner who had “probably” or “definitely” injected drugs; (5) having sex with a partner who “probably” or “definitely” had had male-to-male sexual contact (only female respondents); and (6) having sex with a partner whose HIV status was positive or indeterminate.

### Use of Health Care Services and HIV Testing

Of those participants who reported having visited their health care providers within the last 12 months, only 19.3% (69/358) received an HIV test referral from their health provider. More than half (277/358, 77.4%) of the participants reported having been tested for HIV infection at least once at some point in their lifetime. Of this group, only 30.8% (69/224) of the respondents had tested within the 12 months before the interview. When asked to relate the primary reasons for not getting tested for HIV in the past 12 months, 33.6% (94/280) of the participants’ most frequent answer was being afraid of having HIV*.* Regarding risk perception, 236 (67.4%) out of 350 participants reported perceiving themselves as having a low risk of getting HIV in the next 12 months (Table 1).

### Sociodemographic Characteristics, HIV Testing, High-Risk Sexual Behaviors, and PIHT

Table 1 shows significant differences between the participants who received an HIV test referral from their providers and those who did not. Individuals who reported being aged 30-60 years tended to have a significantly higher prevalence of not receiving an HIV test referral from their providers (*P*=.06). Statistically significant differences between participants who reported having ever been tested for HIV and provider recommendations were observed (*P*<.001). Significant differences were also observed among individuals who had engaged in high-risk sexual behaviors. Those who reported higher risk sexual practices had a lower HIV test referral from their provider (*P*=.050). The difference in the proportion of women who reported engagement in high-risk sexual behaviors achieved marginal significance (*P*=.095). Additionally, women received significantly more HIV test referrals than men, despite no significant differences between the 2 groups in reported high-risk sexual behaviors (post hoc analysis; *P*=.25).

### Factors Associated With PIHT

Multivariate logistic regressions models adjusted for age and sex showed that the estimated prevalence of the participants who received an HIV test referral among those who reported being engaged in high-risk sexual behavior is 41% (PR_a_ .59, 95% CI .39-.91; *P*=.02) lower than those who did not engage in high-risk sexual behavior (Table 2). Moreover, the prevalence of getting an HIV test referral by their health care provider among women who engaged in high-risk sexual practices is 43% (PR_a_ .57, 95% CI .35-.93; *P*=.02) lower than women who reported not being engaged in high-risk sexual behavior. Lastly, the prevalence of receiving a referral for HIV testing by a provider among individuals who reported being tested for HIV in their lifetime is 6 times (CI 95% 2.12-15.28; *P*=.001) the prevalence of getting a referral of HIV testing among those who have not been tested for HIV throughout their life (Table 2).

**Table 2:** Multivariate analysis of variables associated with provider-initiated HIV testing, Puerto Rico National HIV Behavioral Surveillance Cycle 2016 (N=358).

Variable			Received HIV test referral, n (%)		Did not receive HIV test referral, n (%)		Crude prevalence ratio (95% CI)		*P* value		Adjusted prevalence ratio^a^ (95% CI)		*P* value
**Engaged in high-risk sexual behavior^b^ (N=358)^c^**													
	No (n=115)	29 (25.2)		86 (74.8)		Reference		.048		Reference		.02	
	Yes (n=243)	40 (16.5)		203 (83.5)		.65 (.43-.99)				.59 (.39-.91)			
**Women who engaged in high-risk sexual behavior^b^ (n=250)**													
	No (n=85)	22 (25.9)		63 (74.1)		Reference		.09		Reference		.02	
	Yes (n=165)	28 (17)		137 (83)		.66 (.40-1.07)				.57 (.35-.93)			
**Ever tested for HIV (N=358)**													
	No (n=81)	4 (4.9)		77 (95.1)		Reference		.002		Reference		.001	
	Yes (n=277)	65 (23.5)		212 (76.5)		4.75 (1.79-12.65)				5.70 (2.12-15.28)			

^a^Adjusted by sex and age.

^b^Defined as engaging in at least one of the following: (1) any exchange of sex for drugs or money; (2) having sex with more than one sexual partner; (3) having sex with a partner who “probably” or “definitely” had other sex partners, concurrently; (4) having sex with a partner who had “probably” or “definitely” injected drugs; (5) having sex with a partner who “probably” or “definitely” had had male-to-male sexual contact (only female respondents); and (6) having sex with a partner whose HIV status was positive or indeterminate.

^c^Denominators vary because of nonresponse in the form of missing values.

## Discussion

### Principal Findings

This study aimed to examine the characteristics and behaviors of PIHT referrals among heterosexual individuals at increased risk using data from PR-NHBS-HET4. Findings show that most study participants had medical insurance coverage and had visited a health care provider in the past year. Despite their documented access to health services, among those who visited a health care provider within the past 12 months, 80.7% reported they did not receive an HIV test referral from their provider.

### Comparison With Prior Work

The estimated number of participants who reported not receiving an HIV test referral from a health provider in this study is higher than those reported using Virginia’s NHBS data (58%) [8]. This result shows a gap in implementing HIV testing guidelines in health care settings and the need to promote provider HIV screening among heterosexual adults at increased risk of HIV infection [4]. Our study showed that participants who engaged in high-risk sexual behavior were less likely to receive an HIV screening referral. Main barriers including insufficient time, burdensome consent process, lack of knowledge or training, lack of patient acceptance, pretest counseling requirements, competing priorities, and inadequate reimbursement have been identified in a comprehensive literature review [19]. Moreover, similar to other studies, our results showed that many participants perceived themselves to be at low risk of getting infected with HIV [20,21]. This low–risk perception may offer insight into the discrepancy between the number of individuals who seek health services and those who receive an HIV test referral, as having a low perception of risk has been previously linked to a decreased uptake of HIV testing [22,23]. Despite this gap, findings also revealed that women received significantly more HIV test referrals than men, despite no significant differences between the 2 groups in reported high-risk sexual behaviors, which is an observation consistent with the literature [24,25]. This difference between men and women could be partially explained by prenatal care since routine HIV testing during pregnancy has been a long-standing recommendation in the United States [26].

In 2007, the Joint United Nations Program of HIV/AIDS and the World Health Organization issued new guidelines on HIV testing. Even with the rules laid out by international organizations, there have been challenges in the implementation, considering that policies regarding HIV testing are decided by each nation or state individually [27]. Therefore, studies show the variation in HIV test referrals across states and the discrepancy between global expectations and local realities [28,29]. Particularly in Puerto Rico (where either governmental or private insurance covers most of the population) [30], efforts to increase HIV test referrals have recently been supported by public policy. Law Number 45 of May 2016 mandates that HIV testing be referred to patients as part of their routine care. Specifically, individuals aged 13-64 years who are at low risk of infection are to be referred for an HIV test as part of their routine medical testing at least once every 5 years. Individuals at high risk of infection must be referred for an HIV test annually. Additionally, Article 3 of this legislation explicitly states that medical insurance policies, whether private or government-issued, must include an annual HIV test in their primary health insurance coverage [31].

Considering the Puerto Rican public policy regarding HIV test referrals and our findings, we observe a gap that echoes the difficulty in implementing HIV prevention efforts. To bridge this gap, efforts related to HIV stigma education in health care settings [32] and CDC-funded HIV testing events are examples of effective strategies to increase HIV testing uptake. In addition, further identification of predictors of refusal to participate in PIHT can provide more insight into the barriers that limit the scope and referrals of HIV testing in health care settings in Puerto Rico [33].

### Limitations

Among the limitations of this study, we consider the study’s design as one of them. As a cross-sectional study, the impact of the evaluated variables on PIHT over time cannot be assessed. Other limitations include using self-reported information gathered during face-to-face interviews that could have introduced a social desirability bias. The studied population limits the generalizability of the result to other populations with different sociodemographic profiles. Contrarily, however, an advantage of the study is the use of RDS, which has been proven to be a reliable methodology for reaching and recruiting minority populations [34,35]. Recent data for NHBS-HET is available; unfortunately, the variables of interest (eg, HIV risk perception and the high-risk sexual behavior of men and women having unprotected sex with an HIV-positive partner) are not similar in their construction, which limits the opportunity to conduct a merged analysis of different periods. Despite the limitations, this behavioral surveillance system has successfully provided the necessary information for monitoring the implementation of the CDC’s HIV testing guidelines. It has made it possible to better understand the patient-driven HIV testing habits, patient health care access, and risk behaviors of the members of this population, with a particular focus on the Puerto Rican heterosexual individuals at increased risk of HIV infection population. Lastly, compared to other studies, our results are limited due to the scope of PIHT in the literature. Most recent studies evaluated PIHT jointly with referral and counseling, indicating that patients are receptive to this joint approach. These studies had documented the overall high levels of acceptability of PIHT when offered in addition to referrals and counseling [6,7]. As this study was based on PR-NHBS data collection procedures, counseling—which has been included in other studies—was not offered to the extent of providing continual care for the patient.

### Conclusions

In summary, findings from this study show low adherence practices for HIV test referrals in health care settings in Puerto Rico. Increasing adherence to such guidelines among heterosexual individuals at increased risk of HIV infection is critical to ensuring the early diagnosis of unidentified HIV cases, which would lead to better patient care and outcomes that will support the development of more effective awareness and prevention measures [7,36]. Future studies should explore the communication dynamics between the patient and provider in discussing preventative screening. Specifically, a qualitative component should be added to evaluate reasons for opting out of HIV tests, identify barriers to PIHT, and collect information relevant to social factors that affect PIHT (eg, stigma, HIV awareness, the doctor-patient relationship, etc). As policies are enacted to support the implementation of HIV testing referred to patients as part of their routine care, implementation studies should explore the effectiveness of PIHT communication and strategies for increasing adherence to HIV test referral guidelines in different health care settings in Puerto Rico.

## Abbreviations

CDC: Centers for Disease Control and Prevention
NHBS: National HIV Behavioral Surveillance
PIHT: provider-initiated HIV testing
PR-NHBS-HET4: Puerto Rico National HIV Behavioral Surveillance–Heterosexual Cycle 4th round
PR_a_: adjusted prevalence ratio
RDS: respondent-driven sampling
